# Using artificial intelligence to expedite and enhance plain language summary abstract writing of scientific content

**DOI:** 10.1093/jamiaopen/ooaf023

**Published:** 2025-04-03

**Authors:** David McMinn, Tom Grant, Laura DeFord-Watts, Veronica Porkess, Margarita Lens, Christopher Rapier, Wilson Q Joe, Timothy A Becker, Walter Bender

**Affiliations:** Sorcero, East Kilbride G75 8QD, United Kingdom; UCB, Slough SL1 3WE, United Kingdom; Lumanity Communications Inc., Yardley, PA 19067, United States; UCB, Slough SL1 3WE, United Kingdom; UCB, Slough SL1 3WE, United Kingdom; UCB, Slough SL1 3WE, United Kingdom; Lumanity Communications Inc., Yardley, PA 19067, United States; Lumanity Communications Inc., Yardley, PA 19067, United States; Sorcero, Washington, DC 20005, United States

**Keywords:** artificial intelligence (AI), health communication, health literacy, plain language summary (PLS)

## Abstract

**Objective:**

To assess the capacity of a bespoke artificial intelligence (AI) process to help medical writers efficiently generate quality plain language summary abstracts (PLSAs).

**Materials and Methods:**

Three independent studies were conducted. In Studies 1 and 3, original scientific abstracts (OSAs; *n* = 48, *n* = 2) and corresponding PLSAs written by medical writers versus bespoke AI were assessed using standard readability metrics. Study 2 compared time and effort of medical writers (*n* = 10) drafting PLSAs starting with an OSA (*n* = 6) versus the output of 1 bespoke AI (*n* = 6) and 1 non-bespoke AI (*n* = 6) process. These PLSAs (*n* = 72) were assessed by subject matter experts (SMEs; *n* = 3) for accuracy and physicians (*n* = 7) for patient suitability. Lastly, in Study 3, medical writers (*n* = 22) and patients/patient advocates (*n *= 5) compared quality of medical writer and bespoke AI-generated PLSAs.

**Results:**

In Study 1, bespoke AI PLSAs were easier to read than medical writer PLSAs across all readability metrics (*P* <.01). In Study 2, bespoke AI output saved medical writers >40% in time for PLSA creation and required less effort than unassisted writing. SME-assessed quality was higher for AI-assisted PLSAs, and physicians preferred bespoke AI-generated outputs for patient use. In Study 3, bespoke AI PLSAs were more readable and rated of higher quality than medical writer PLSAs.

**Discussion:**

The bespoke AI process may enhance access to health information by helping medical writers produce PLSAs of scientific content that are fit for purpose.

**Conclusion:**

The bespoke AI process can more efficiently create better quality, more readable first draft PLSAs versus medical writer-generated PLSAs.

## Background 

Clear communication of scientific advancements through publication is an essential part of the scientific process. Studies have also shown that health literacy is strongly associated with patient outcomes.^1^^,^^2^ Therefore, access to scientific information that is comprehensible to lay audiences has the potential to enhance patient adherence to medical guidance, empower patients and their advocates to participate in decision-making, and foster greater equity in health care.^3^ To maximize comprehension of scientific information across a wide audience while retaining essential medical content, the National Institutes of Health and American Medical Association recommend that best practices for plain language and materials used for patient medical education be written at a 6th- to 8th-grade reading level, which corresponds to ages 11-13 (approximately 6-8 years of education).^4^^,^^5^ Recognizing the need for transparency in medical communications, regulatory agencies now require lay summaries of clinical trial results and risk management plans.^6^

Plain language summaries (PLSs)—simplified versions of scientific content written in easy-to-understand language for non-experts—are a tool that can bridge the gap between complex medical information and lay audiences. PLSs aim to ensure that health information is accessible, comprehensible, and conducive to informed decision-making, thereby promoting basic understanding, effective communication, and critical thinking with the goal of promoting health literacy.^7–13^ A substantial body of evidence indicates that current PLSs often fail to meet the standards required to be accessible to the general public, suggesting that scientific communicators have struggled to fulfill the potential of PLSs.^9^^,^^14–22^ Therefore, there is still a need to aid authors and medical writers in generating PLS content, such as plain language summary abstracts (PLSAs), that are more suitable for lay audiences.

Generative artificial intelligence (AI) has been touted as a revolutionary tool with the potential to promote rapid development of scientific information at an accessible reading level.^23^ While many publicly available consumer-grade AI tools exist, caution should be exercised if they are to be used in developing PLS content, as they are prone to hallucinations and unfounded assertions,^24^ and interactions with these platforms may not be private. As content accuracy, integrity, and confidentiality are paramount in scientific communication, there is an unmet need for tailored AI tools that offer task-specific workflows for the generation of high-quality content while ensuring hallucination mitigation, content validation, and data security.

## Objective

Herein, 3 independent studies were conducted to assess the capacity of a purpose-built bespoke AI process to produce readable PLSAs, as well as the impact of the bespoke AI process on the efficiency of PLSA development.

## Methods

### AI PLSA generation process

In these studies, 2 approaches for utilizing AI to generate PLSAs were applied: a non-bespoke AI approach and a proprietary, purpose-built bespoke AI approach.

The non-bespoke AI approach involved pasting the text of an original scientific abstract (OSA) into the interface of a publicly available large language model (LLM) tool (Chat Generative Pre-Trained Transformer [ChatGPT], model 3.5T^25^) with the following prompt: “Summarize the following text at the level of a Grade 6 student: [*OSA text inserted*].”

The bespoke AI process combined natural language processing and LLMs (GPT 3.5T, Biomedical Bidirectional Encoder Representations from Transformers [BioBERT],^26^ Bidirectional and Auto-Regressive Transformer [BART],^27^ Scientific Bidirectional Encoder Representations from Transformers [SciBert],^28^ and Disease Ontology Indentifiers (DOID),^29^ and National Cancer Institute Thesaurus [NCIT]^30^ ontologies) in a 3-step process: (1) a classifier extracted structure from the source document, (2) an LLM generated a summary of each element within that structure, and (3) the output of the LLM was validated using language and ontological models specific to the life sciences. The bespoke AI process was developed and refined at the beginning of Study 1, and the refined process was used to generate all the PLSAs reported herein. An example of the output of the bespoke AI process and a block diagram describing its operation are shown in Figures S1 and S2.

#### PLSA definition

Nomenclature varies with respect to the types and styles of plain/lay language documents, and arguably there are no consensus definitions. For the purposes of this study, we define a PLSA as a short document (no more than 1 page) that provides sufficient information for the reader to grasp the background, methods, results, and conclusion of a study, without going into a great amount of detail (similar to that of a scientific abstract, but in more accessible language). On the other hand, a PLS is a longer document of 2-5 pages in length that provides a comprehensive description of the source content (eg, a published manuscript or clinical trial report). In this study, we focus on generation of the shorter PLSAs, rather than the longer PLSs.

### Study 1: PLSA readability and subject matter expert quality assessment

**Figure 1.**
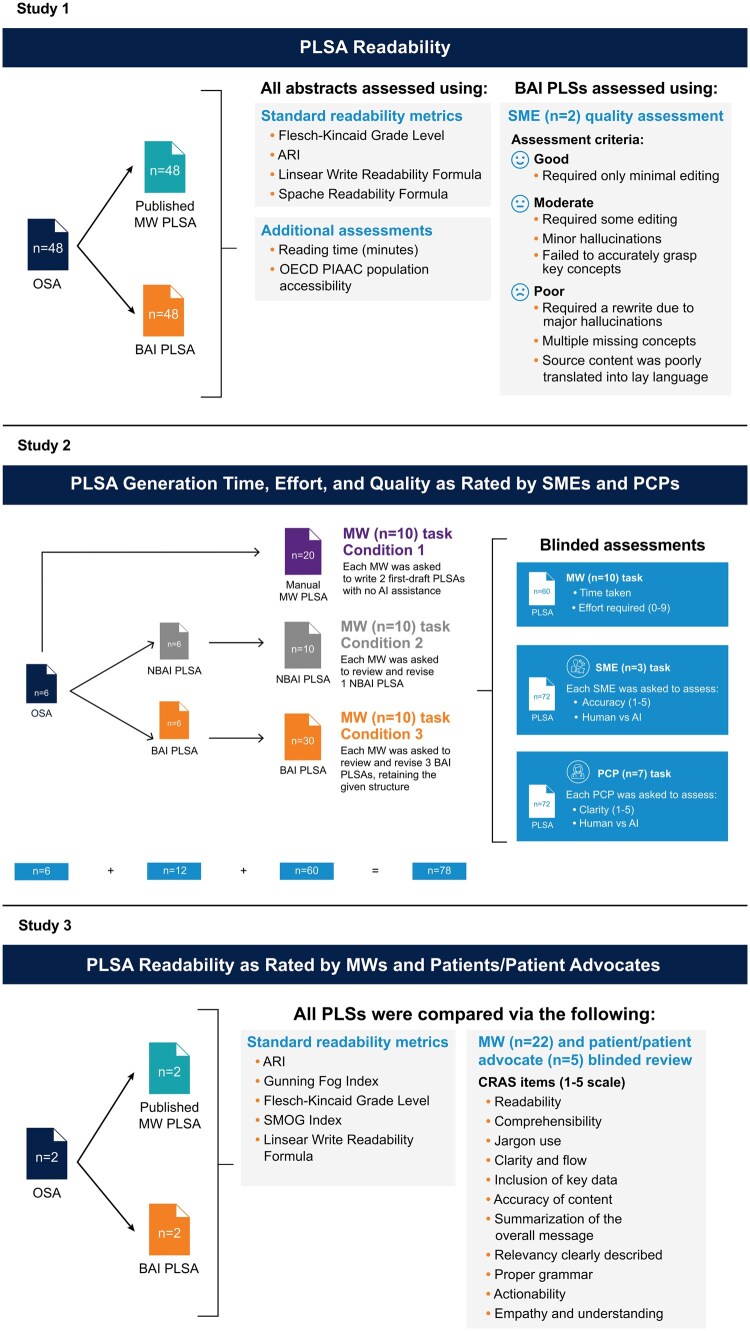
Study designs. AI = artificial intelligence; ARI = automated readability index; BAI = bespoke artificial intelligence; CRAS = comprehensive readability assessment scale; MW = medical writer; NBAI = non-bespoke artificial intelligence; OECD = organization for economic co-operation and development; OSA = original scientific abstract; PCP = primary care physician; PIAAC = programme for the international assessment of adult competencies; PLSA = plain language summary abstract; SME = subject matter expert; SMOG = simple measure of gobbledygook.

Study 1 compared the readability of published OSAs to their accompanying published PLSAs written by medical writers and to PLSAs generated by the bespoke AI process.^29^ The study design is outlined in Figure 1.

#### PLSA identification

A total of 50 PLSAs written by medical writers published in 2022 and 2023 were identified and selected via PubMed using the search term “plain language summary.” The target of 50 was pragmatically chosen as a manageable number to assess. The therapy areas and publication sources of these abstracts are shown in Table S1. For each medical writer PLSA, the corresponding OSA was retrieved. For 2 PLSAs, the corresponding OSA could not be obtained, leaving a total of 48 PLSAs and corresponding OSAs for assessment. A bespoke AI PLSA was generated for each OSA, using the OSA as the source document.

#### Assessment

Readability scores were calculated for all OSAs, medical writer PLSAs, and bespoke AI PLSAs using 5 standard readability metrics (Flesch-Kincaid Grade Level, Flesch Reading Ease, Automated Readability Index [ARI], Linsear Write Readability Formula, and SPACHE Readability Formula).^31–35^ Mean readability scores for the OSAs, medical writer PLSAs, and bespoke AI PLSAs were then mapped onto a measure of population literacy based on the Organisation for Economic Co-operation and Development Programme for the International Assessment of Adult Competencies (PIAAC)^36^ data for the United States (*n* = 3892)^37^ as a metric of population-level accessibility. Reading time was calculated using a modified version of the Demberg and Keller algorithm, which takes into account time required to scan a text, time required to comprehend the scanned text, and grade level associated with the text.^38^

Finally, 2 subject matter experts (SMEs) (1 pharmacy-trained medical affairs professional and 1 plain language content expert) conducted a quality analysis of the bespoke AI PLSAs using the following scale: 1 = “Poor,” which required a rewrite due to major hallucinations, had multiple missing concepts, or had poor translation of the source content into lay language); 3 = “Moderate,” which required some editing, had minor hallucinations, or failed to accurately grasp key concepts from the source document in places; and 5 = “Good,” which required only minimal editing before being classified as a suitable first draft. Both SMEs assessed all 48 PLSAs and the mean quality assessment score was calculated.

### Study 2: PLSA generation time, effort, and quality as rated by SMEs and physicians

Study 2 assessed whether providing medical writers with first-draft PLSAs developed by generative AI (non-bespoke AI and bespoke AI processes) produced time- and effort-saving value compared with fully manual (ie, no AI assistance) PLSA development.^39^ Six OSAs on rare neurological and rheumatological diseases were used in this study (1 phase 2 trial; 4 phase 3 trials; and 1 survey-based study; Figure 1). The OSAs were used as source material for medical writers to manually draft PLSAs and as input for PLSA development using both the non-bespoke AI and bespoke AI approaches. The manually developed, non-bespoke AI-developed, non-bespoke AI-assisted, bespoke AI-developed, and bespoke AI-assisted PLSAs were compared in terms of their accuracy according to 3 SMEs (different SMEs than those in Study 1) and clarity according to 7 primary care physicians (PCPs).

#### Medical writer task

Ten medical writers (4 from the United States, 5 from the United Kingdom, and 1 from France) who were experienced in developing plain language content took part in the study. The medical writers provided written informed consent and were paid at fair market value for their time. Each medical writer was asked to write a total of 6 first-draft PLSAs to a standard that they considered to be a “good first draft” under 3 different conditions. Condition 1: provided 2 separate OSAs and asked to manually develop a 400- to 500-word PLSA for each. Condition 2: provided with a non-bespoke AI PLSA and asked to review/revise the content to generate 1 PLSA structured according to any format that they desired. Condition 3: provided 3 separate bespoke AI PLSAs and asked to review/revise the content of each to generate 3 separate PLSAs, retaining the structure of the bespoke AI PLSAs initially provided. For conditions 2 and 3, medical writers were also given the OSAs as a reference. For all conditions, the medical writers were further instructed that PLSAs should be text only, with no visuals or graphical elements, and that they were not to use any form of generative AI tool to assist with developing the PLSAs outside of the text they may have initially been provided. Medical writers were not provided with a suggested target grade level for the PLSAs. Medical writers were given 16 hours to develop/finalize all 6 PLSAs across the 3 conditions.

Abstracts were randomly assigned to each task condition; task order was also randomly assigned to control for any potential learning or ordering bias. Medical writers were blinded to the conditions for the AI-assisted drafts. After completing each PLSA, they were asked to rate:

- The time taken (in minutes) to complete each PLSA; and
- The mental effort required to complete each PLSA, based on a 9-point cognitive load Likert scale (higher scores reflect more mental effort).^40^

#### SME assessment

Blinded SMEs (*n* = 3; medical publication professionals) were asked to review PLSAs that were AI generated without any medical writer intervention (non-bespoke AI = 6; bespoke AI = 6) and those developed or finalized by the medical writers (*n* = 60; conditions 1-3), for a total of 72 PLSAs. Each SME reviewed 24 PLSAs in a randomized order, blinded to the development condition. They were asked to assess the PLSAs based on (1) the content accuracy of the PLSA versus the OSA (1-5; higher scores reflect better accuracy) and (2) speculation of whether the PLSA was manually developed by a medical writer or drafted using generative AI.

#### PCP assessment

Blinded PCPs (*n* = 7; 3 from the United States and 4 from the European Union) scored the PLS abstracts (*n* = 72; 5 PCPs reviewed 10 PLSAs and 2 PCPs reviewed 11 PLSAs) in randomized order using a 5-point Likert scale to gauge clarity and suitability for effectively communicating research findings to patients in a comprehensible manner (higher scores reflected better clarity). PCPs were also asked to speculate whether the PLSA was manually developed by a medical writer or drafted using generative AI.

#### Additional assessments

The readability of the OSA (*n* = 6) and the medical writer PLSAs, non-bespoke AI PLSAs, and bespoke AI PLSAs (*n* = 72; total *n* = 78) was measured using the Flesch-Kincaid Grade Level scale. The Levenshtein distance^41^ (ie, minimum number of single-character edits [insertions, deletions, or substitutions] required to change between the source [non-bespoke AI PLSA or bespoke AI PLSA] and the final draft of the medical writer PLSA) was also calculated to quantify the extent of medical writer intervention required to revise the bespoke AI- and non-bespoke AI-generated PLSAs to a first draft they considered to be of “good quality.”

### Study 3: PLSA readability as rated by medical writers and patients/patient advocates

In the final study, 2 open-access clinical trial articles published between 2021 and 2022 from distinct therapeutic areas (oncology and infectious disease) with associated medical writer PLSAs were identified via PubMed.^42^^,^^43^ The medical writer PLSAs served as the comparator. The same bespoke AI process from Studies 1 and 2 was used to develop 2 separate bespoke AI PLSAs, with the OSA serving as input (Figure 1).

All PLSAs were scored for readability using 5 standard readability metrics: ARI, Gunning Fog Index, Flesch-Kincaid Grade Level, Simple Measure of Gobbledygook (SMOG) Index, and Linsear Write Readability Formula. PLSAs were also scored by blinded reviewers (medical writers [*n* = 22] and patients/patient advocates [*n* = 5]) using a custom 5-point (1 = very poor; 2 = poor; 3 = fair; 4 = good; 5 = excellent) comprehensive readability assessment scale (CRAS) that evaluated 11 items: readability, comprehensibility, jargon usage, clarity and flow, inclusion of key data, accuracy of content, summarization of the overall message, relevancy clearly described, proper grammar, actionability, and empathy and understanding.

Reviewers were instructed to read the OSA and the 2 corresponding PLSAs (ie, the medical writer PLSAs and bespoke AI PLSAs) and rank CRAS items for the PLSAs. For the first PLSA, the medical writer PLSA was provided first, followed by the bespoke AI PLSA. For the second PLSA, the bespoke AI PLSA was provided first, followed by the medical writer PLSA.

Content accuracy for the PLSAs was evaluated by an unblinded medical writer trained in hematology and infectious diseases.

### Ethical approval

As the study exclusively utilized publicly available PLSAs and no participant information was captured, stored, or analyzed, ethical approval was neither sought nor obtained for this investigation.

### Statistical analyses

Inferential statistical analyses were conducted for readability scores and reading time in Study 1 and for PLSA generation time in Study 2. Mean differences were compared using one-way analysis of variance (ANOVA) with Bonferroni-corrected pairwise comparisons at an alpha level of *P* =.05. Relevant assumptions for ANOVA were met for the analysis of readability scores. For the analysis of reading time and PLSA generation time, homogeneity of variance was not met. Consequently, the data were log transformed before running the ANOVA for these analyses. For the assessment of readability scores and reading time in Study 1, based on 3 groups of 48 PLSAs, a power of >99% to detect a large effect size (Cohen’s *f* = 0.4) at an alpha level of *P* =.05 was achieved. For the assessment of PLSA generation time in Study 2, a power of 79% for detecting a large effect size (Cohen’s *f* = 0.4) at an alpha level of *P* =.05 between the manual- and bespoke-AI approaches was achieved.

## Results

### Study 1: PLSA readability

**Figure 2.**
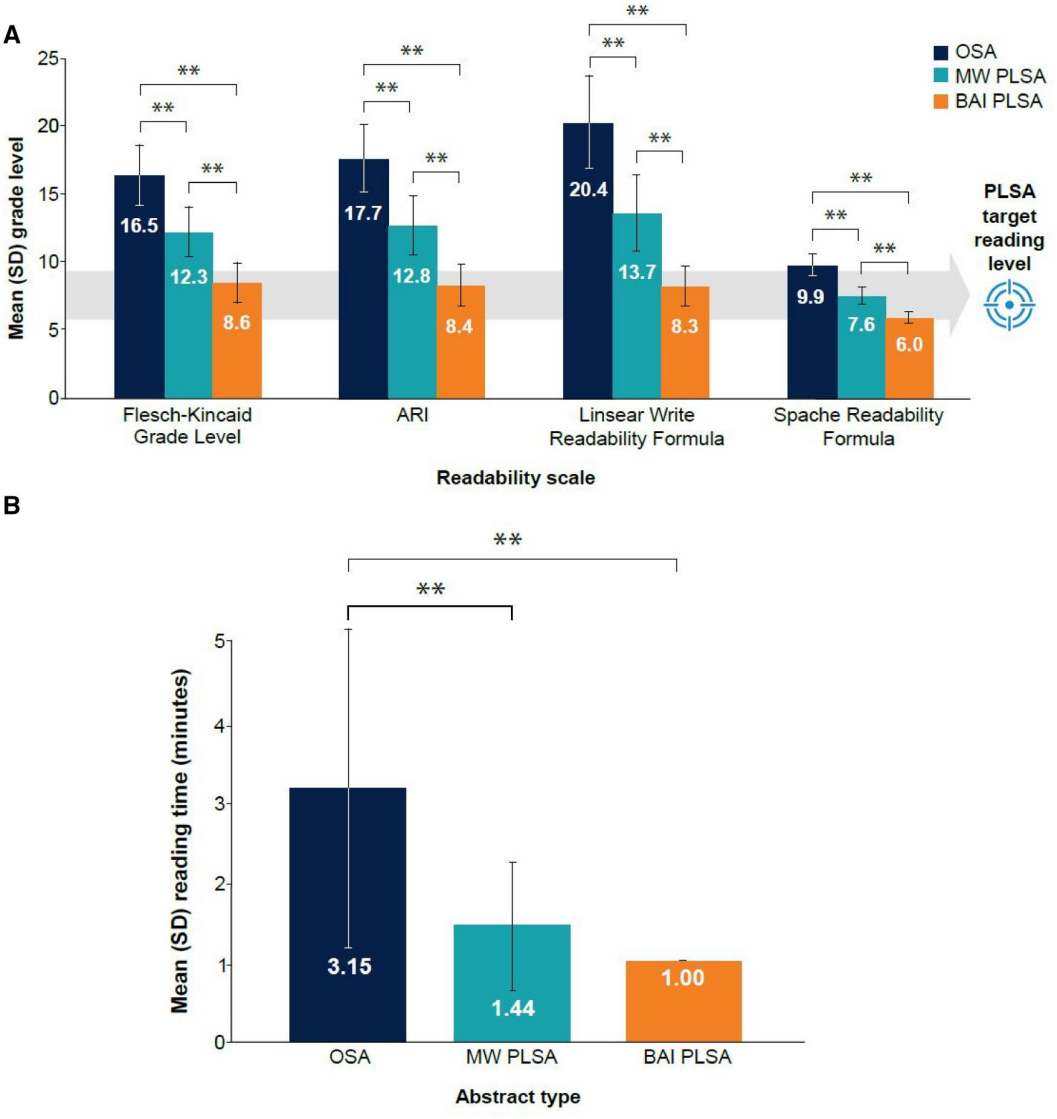
Study 1 outcomes: (A) mean grade-level readability and (B) reading time by PLSA type. ANOVA = analysis of variance; ARI = automated readability index; BAI = bespoke artificial intelligence; MW = medical writer; OSA = original scientific abstract; PLSA = plain language summary abstract; SD = standard deviation. ANOVA with Bonferroni pairwise comparisons. ANOVA for reading time conducted using Log-transformed data. **P<.001. The PLSA target reading level of 6th- to 8th-grade reflects the recommendation by the American Medical Association and National Institutes of Health.^4^^,^^5^

Mean reading scores/US grade level for the OSAs and the medical writer and bespoke AI PLSAs are shown in Figure 2A. For each scale, omnibus ANOVA results demonstrated significant differences in readability between PLSA types (*P* <.05 for all omnibus tests). The Bonferroni-corrected pairwise comparisons within each scale showed significant differences when comparing the medical writer PLSAs and bespoke AI PLSAs (*P* <.01); medical writer PLSAs had higher grade-level scores versus bespoke AI PLSAs. Overall, mean Flesch Reading Ease score for the OSAs was 17 (very confusing), suitable for a “college graduate” audience (aged approximately 20+ years). Medical writer PLSAs were scored as 12 (difficult), suitable for a “college” audience (aged approximately 17-20 years), while bespoke AI PLSAs were scored as 9 (standard), suitable for an “8th- to 9th-grade” audience (aged approximately 13-15 years).

Abstract reading times ranged from 1 to 3.15 minutes (Figure 2B). Omnibus ANOVA analysis demonstrated significant between-group differences in reading times (*F* = 61.93; *P* <.001). Bonferroni-corrected pairwise comparisons indicate that mean reading times for medical writer PLSAs (1.44 minutes) and bespoke AI PLSAs (1.00 minute) were significantly lower (*P* <.001 for each comparison) than mean reading time for the OSAs (3.15 minutes).

**Figure 3.**
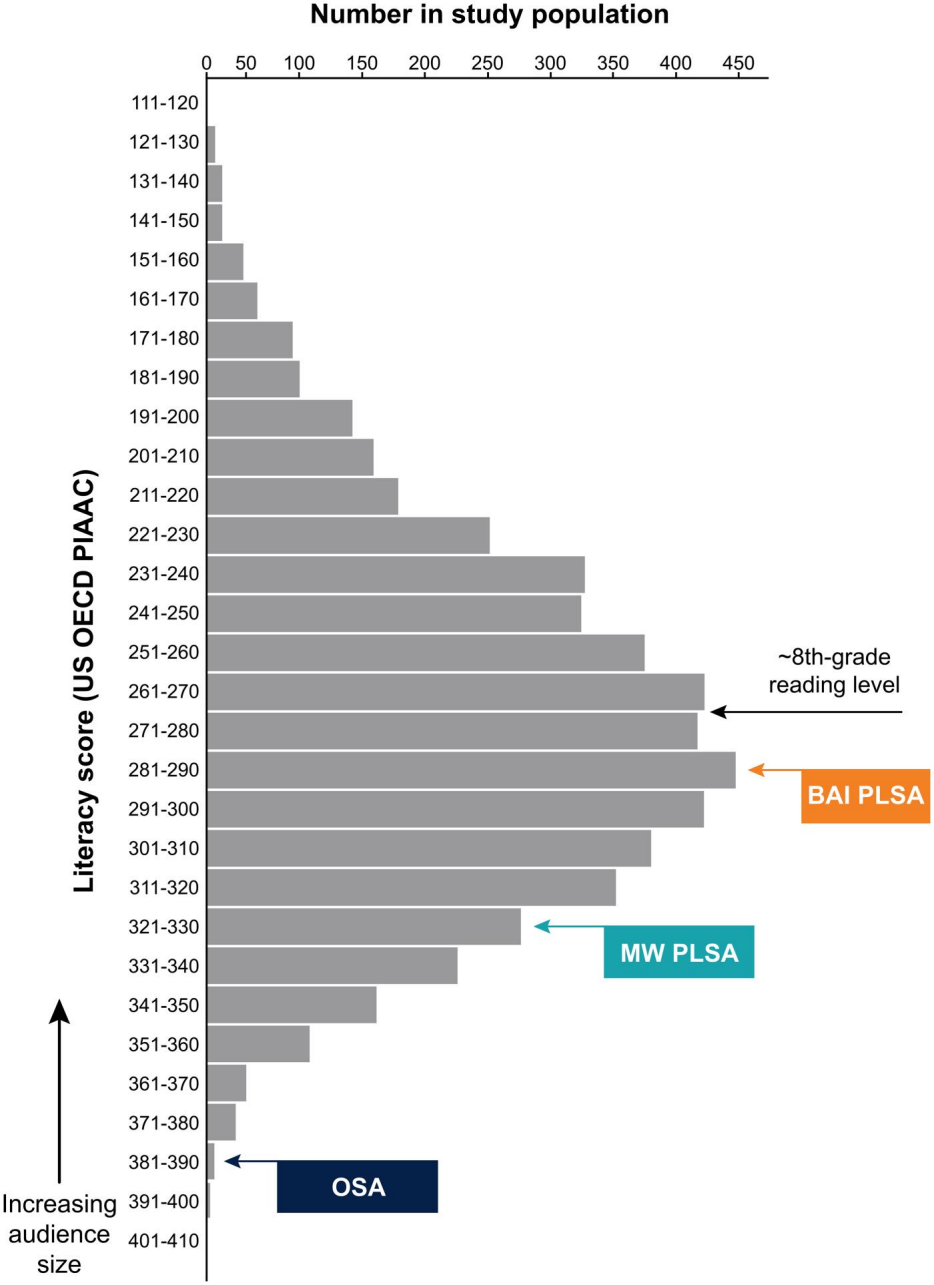
Study 1 outcome: PLSA accessibility as measured by US OECD PIAAC population literacy data (*n* = 3892). BAI = bespoke artificial intelligence; MW = medical writer; OECD = organization for economic co-operation and development; OSA = original scientific abstract; PIAAC = programme for the international assessment of adult competencies; PLSA = plain language summary abstract.

Finally, when OSAs, medical writer PLSAs, and bespoke AI PLSAs were mapped onto the PIAAC United States population-level distribution of literacy abilities (based on grade-level readability scores), the bespoke AI PLSAs were deemed accessible to the largest percentage of the population (∼50%), followed by medical writer PLSAs (∼10%) and OSAs (∼1%; Figure 3).

Using the 5-point scale, SME analysis of the quality of 48 bespoke AI PLSAs resulted in a mean (standard deviation [SD]) of 4.55 (0.98). The intraclass correlation coefficient (ICC) was 0.79 (confidence interval [CI] 0.67-0.89), demonstrating high inter-rater reliability. SME analysis of medical writer PLSAs was not performed.

### Study 2: PLSA generation time, effort, and quality as rated by SMEs and physicians

**Figure 4.**
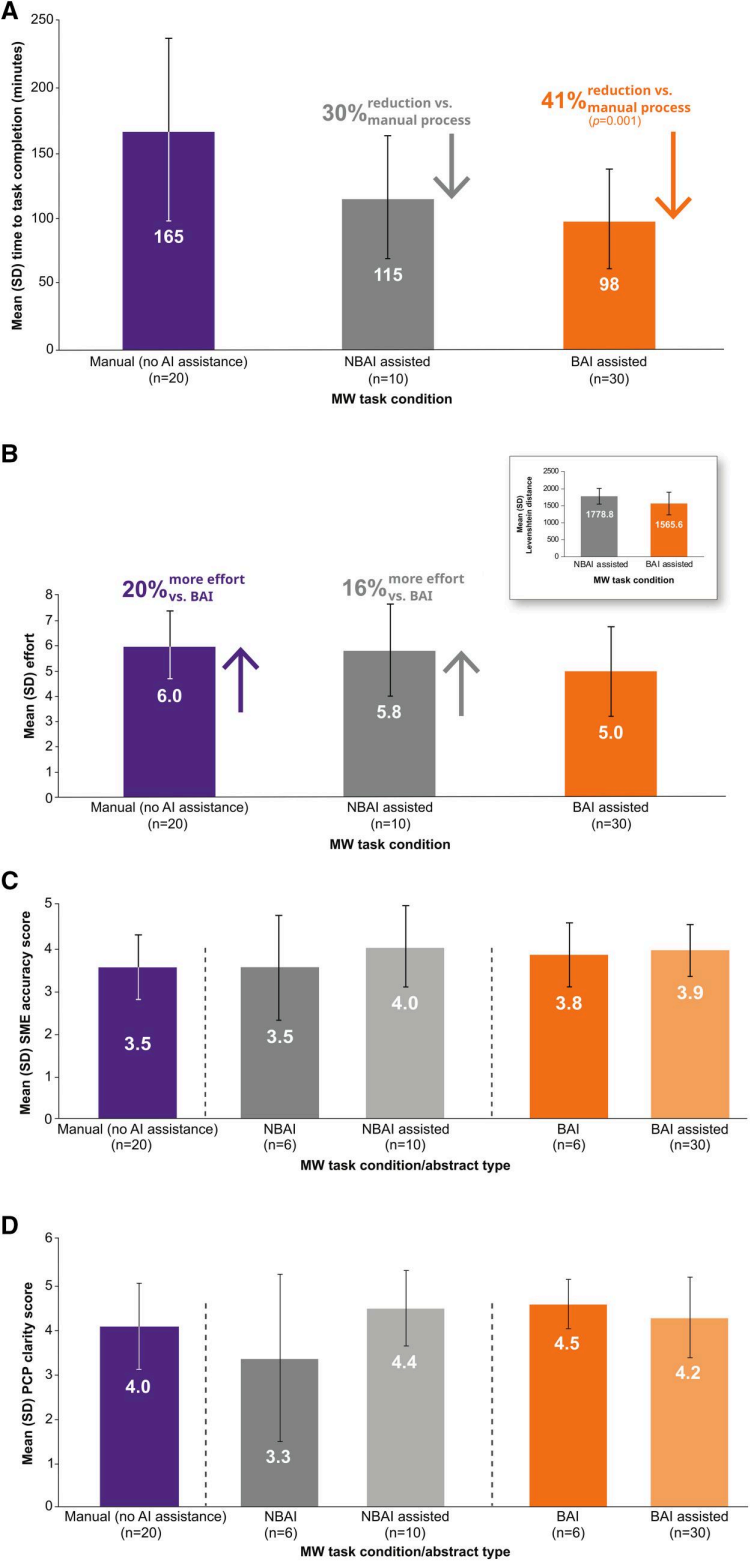
Study 2 outcomes: (A) time to complete task by condition, (B) effort to complete task by condition, (C) SME-assessed accuracy by PLSA type, and (D) PCP clarity assessment by PLSA type. AI = artificial intelligence; ANOVA = analysis of variance; BAI = bespoke artificial intelligence; MW = medical writer; NBAI = non-bespoke artificial intelligence; PCP, = primary care physician; PLSA = plain language summary abstract; SD = standard deviation; SME = subject matter expert. ANOVA for time to task completion (A) was conducted using Log-transformed data. For (C) and (D), higher scores reflect better accuracy and clarity, respectively.

Mean (SD) time to complete manual (no AI assistance), non-bespoke AI-assisted, and bespoke AI-assisted PLSAs was 165 (68.66), 115 (46.71), and 98 (37.33) minutes, respectively, reflecting significant between-group differences (*F* = 7.48; *P* <.001; Figure 4A). A reduction of 30.3% in the amount of time it took to develop a first-draft PLSA was observed when medical writers were provided with the non-bespoke AI PLSAs compared with the fully manual process (mean difference = 50 minutes; *P* >.05); 40.6% reduction was observed when medical writers were provided with the bespoke AI PLSAs (mean difference = 67 minutes; *P* =.001). When comparing the manually developed, non-bespoke AI-assisted, and bespoke AI-assisted PLSAs, mean effort to complete the task was 6.0, 5.8, and 5.0 respectively (scale of 0.0-9.0; Figure 4B); both manually developed and non-bespoke AI-assisted processes required more effort than the bespoke AI-assisted process (20.0% and 16.0%, respectively). This was supported by Levenshtein distance measurements, which showed that medical writers made more changes to the text when starting with non-bespoke AI PLSAs (mean changes [SD] = 1778.8 [230.5]) versus the bespoke AI PLSAs (mean changes [SD] = 1565.6 [333.0]).

Using a 5-point scale, the mean (SD) SME accuracy scores were similar for non-bespoke AI-assisted PLSAs (4.0 [0.94]) and bespoke AI-assisted PLSAs (3.9 [0.60]), both of which were higher than accuracy scores for manual (no AI assistance) PLSAs (3.5 [0.76]; Figure 4C). SMEs were unable to reliably identify the development process as human versus AI, identifying the correct process 55.6% of the time.

Using another 5-point scale, mean (SD) PCP clarity scores for PLSAs developed manually or through non-bespoke AI-assisted or bespoke AI-assisted processes were 4.0 (0.97), 4.4 (0.84), and 4.2 (0.91), respectively (Figure 4D). PCPs correctly identified the development process as human or AI assisted 61.1% of the time.

### Study 3: PLSA readability and CRAS scores by medical writers and patients

**Figure 5.**
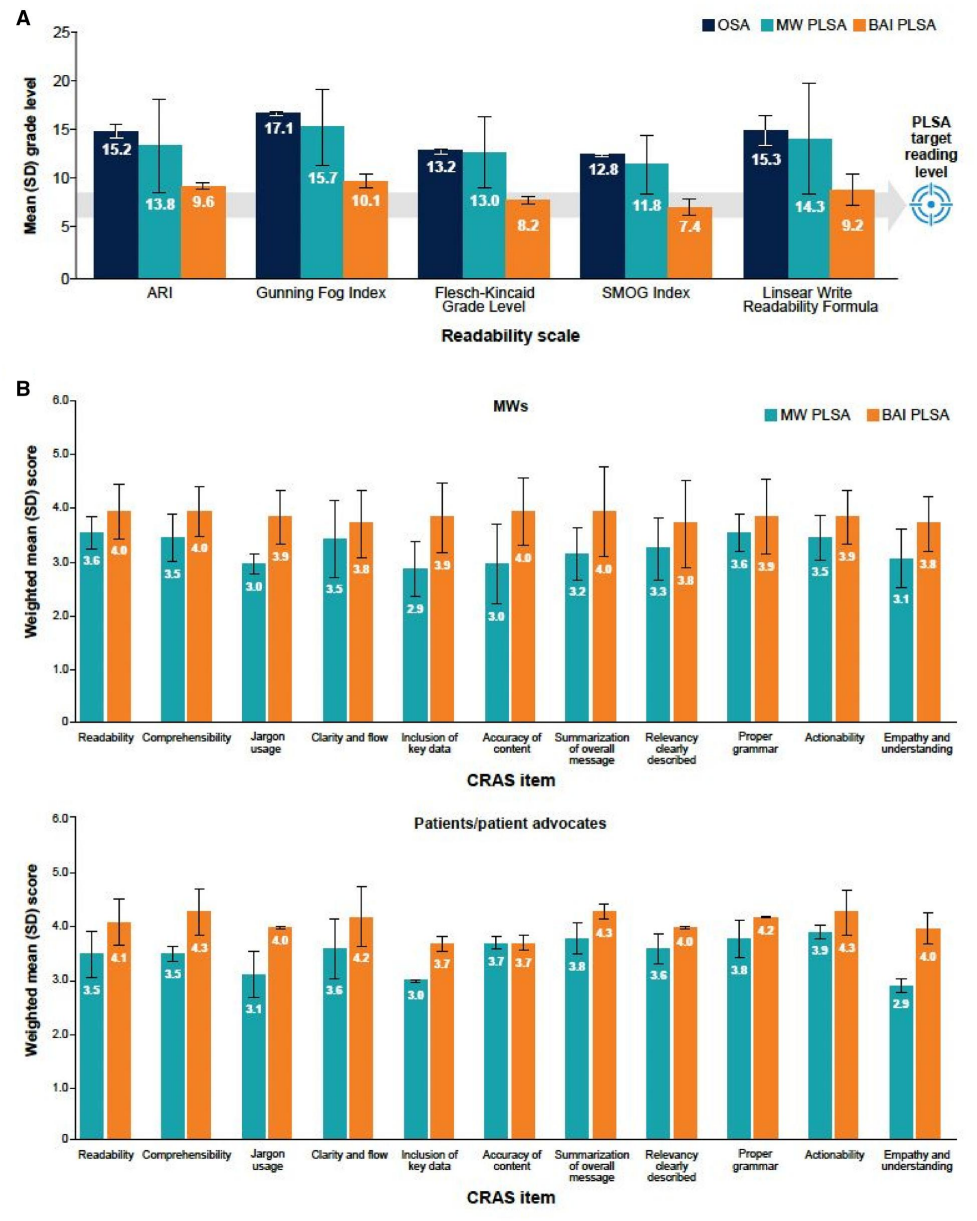
Study 3 outcomes: (A) grade-level readability scores by PLSA type and (B) medical writer (top)- and patient/patient advocate (bottom)-assessed qualitative readability metrics by PLSA type. ARI = automated readability index; BAI = bespoke artificial intelligence; CRAS = comprehensive readability assessment scale; MW = medical writer; OSA = original scientific abstract; PLSA = plain language summary abstract; SD = standard deviation; SMOG = simple measure of gobbledygook. The PLSA target reading level of 6th- to 8th-grade reflects the recommendation by the American Medical Association and National Institutes of Health.^4^^,^^5^

Using standard metrics, overall mean (SD) grade-level readability score (averaged across all 5 readability indices) for medical writer and bespoke AI PLSAs were 13.7 (3.5) and 8.9 (1.2), respectively. Comparisons for individual readability scales are shown in Figure 5A.

When evaluated by medical writers (*n* = 22), overall mean (SD) CRAS scores were 3.3 (0.5) for medical writer PLSAs and 3.9 (0.7) for bespoke AI PLSAs, with higher scores reflecting better quality (Figure 5B). When comparing medical writer with bespoke AI PLSAs, the largest differences were observed for jargon usage (3.0 [0.2] versus 3.9 [0.5]), inclusion of key data (2.9 [0.5] versus 3.9 [0.7]), and accuracy of content (3.0 [0.8] versus 4.0 [0.7]), each favoring the bespoke AI. The ICC for the CRAS scale was 0.63 (CI, 0.57-0.68), demonstrating moderate reliability.

When evaluated by patients/patient advocates (*n* = 5), overall mean (SD) CRAS scores were 3.5 (0.3) and 4.1 (0.2) for medical writer and bespoke AI PLSAs, respectively, with the most pronounced differences in scores for jargon usage (3.1 [0.4] versus 4.0 [0.0]) and empathy and understanding (2.9 [0.1] versus 4.0 [0.3]; Figure 5B), each favoring the bespoke AI. The ICC for the CRAS scale was 0.72 (CI 0.64-0.78), demonstrating moderate reliability.

For both reviewer groups, CRAS items for bespoke AI PLSAs were rated more consistently as “good” or “excellent,” while medical writer PLSAs were more consistently rated as “fair.”

One of the Study 3 abstracts was related to the topic of COVID-19. For this abstract, the accuracy item scores for the medical writer PLSA and bespoke AI PLSA were similar when rated by both medical writers (3.6 versus 3.5, respectively) and patients/patient advocates (3.6 versus 3.6, respectively). Similar content accuracy errors were present in both the medical writer PLSA and bespoke AI PLSA. For the medical writer PLSA, the phrase “COVID-19 virus” was used to describe SARS-CoV-2 even though the phrase should have more accurately been written as “COVID-19, which is caused by the SARS-CoV-2 virus.” In the bespoke AI PLSA, COVID-19 was described as “another type of virus” in a manner that suggested it was distinct from SARS-CoV-2.

## Discussion

Taken together, these 3 independent studies show that generative AI assists medical writers in producing high-quality PLSAs more quickly and efficiently than traditional manual drafting approaches. Specifically, Study 1 demonstrated that the bespoke AI process resulted in better readability scores than PLSAs developed by medical writers, facilitating accessibility to a larger percentage of the adult population. Study 2 showed that a bespoke AI-assisted approach reduced time and effort of PLSA generation while maintaining accuracy and clarity. Finally, Study 3 reinforced that a bespoke AI process results in more readable PLSAs, as demonstrated by both quantitative readability metrics and qualitative assessment by medical writers and patients/patient advocates.

While the benefits of utilizing bespoke AI for generating PLSAs shown herein are compelling, there are some limitations to these studies. While we successfully applied the bespoke AI process to a broad range of therapy areas and measured results across a variety of metrics, our sample size, especially in Study 3, was small. In Study 2, medical writers were not given a specific target grade-level reading score; rather, they were simply instructed to write in plain language. Asking medical writers to aim for a specific grade level in terms of readability could have influenced their output. Finally, the SME scale used in Study 1, the SME and PCP scales used in Study 2, and the CRAS used in Study 3 have not been previously validated. Although inter-rater reliability in studies 1 and 3 was moderate to high, there is always a degree of individual bias that could influence results and the findings of this study should be interpreted accordingly.

### Keeping humans in the loop

During the course of conducting these studies, we observed a number of idiosyncrasies related to LLMs that support the need for human oversight. While generative AI offers promise in facilitating PLSA development, a degree of caution should still be exercised. LLMs are, by their design, predictive and prone to adding additional material to the end of an assertion. This is particularly problematic for the “Background” and “Methods” sections of summaries, where there is a tendency for models to project results into sections where they have not been reported.

Additionally, model bias can be difficult, if not impossible, to overcome. For example, in an article about breast cancer, the LLM insisted on referring to women, even though men can also be diagnosed with breast cancer. No quantity of prompt engineering, modifying the temperatures (a model parameter that influences outputs), or adjusting other model weights could dissuade the model from this assertion. While the example above could reasonably be explained by a not-unexpected model association between women and breast cancer, sometimes the origins of hallucinations are not obvious. In one example, a summary of a description of the population in an obesity survey was described as a survey about long COVID. Regardless of the temperature setting in the model, this hallucination persisted.

Some models appear to be programmed with filtering mechanisms to handle sensitive or controversial topics, such as conspiracy theories. As an example, when asked to translate the journal article title “Randomized controlled trial of favipiravir, hydroxychloroquine, and standard care in patients with mild/moderate COVID-19 disease” into lay language, some LLMs returned nothing. It is likely that the term “hydroxychloroquine” was flagged as a keyword in these models, as it was subject to numerous conspiracy theories. When the word “hydroxychloroquine” was replaced with “paxlovid,” the LLM returned results.

Some of the language output from LLMs can be potentially misleading. For example, one popular LLM used the term “fake medicine” in place of placebo. Other language outputs by LLMs were not deemed suitable for their intended audience. As an example, to maintain scientific accuracy, descriptions of adverse events and endpoints were occasionally not as “plain” as preferred and could be viewed as overgeneralized. To address this, technical terms for adverse events could be followed by a plain language translation. Similarly, trial endpoints could be replaced with simplified descriptions, provided this does not negatively impact the flow/grammar of a sentence or diminish the accuracy. Finally, in some cases, LLMs add emphasis where it may not be appropriate, such as adding the word “only” when reporting results in a manner that implies significance (eg, “survival was 10 months in group 1, but *only* 3 months in group 2”). Overall, these examples reinforce the importance of keeping humans in the loop when using LLM-based tools to generate PLS content.

### Research in context and future directions

Herein, we have established clear benefits of a bespoke AI approach to generating PLSAs. These findings build on recent work demonstrating similar benefits using non-bespoke publicly available LLM interfaces for comparable use cases, including generation of lay abstracts,^44^ plain language inpatient discharge summaries,^45^ layperson summaries,^46^ primary care decision aids,^47^ plain language radiology reports,^48^ and patient information sheets.^49^ Future work could evaluate the application of bespoke AI for not only these use cases but also other types of scientific communications, including first-draft scientific abstracts and manuscripts, press releases, social media posts, image-based content, and video. Additionally, in these studies, we did not evaluate PLSA generation with a variety of different LLMs, and model development is rapidly changing. Thus, there may be room for further improvement in the bespoke AI process by using a combination of alternative models.

### Using AI assistance to support PLSA best practice

Both the bespoke and non-bespoke processes can be readily incorporated into the workflow of researchers and medical writers using the approaches outlined in the “Methods” section of this paper and Figure S2.

### Improved metrics

While our findings demonstrate that the bespoke AI approach produced PLSAs that were easier to read, the measures of readability used herein were based on standard reading metrics. Such metrics use formulas that quantify readability by accounting for the average number of words per sentence and syllables per word. These metrics make no accommodation for the highly specialized vocabulary used in medical publishing. In this context, reading time calculations may be similarly imprecise. Hence, there is an unmet need to develop readability metrics that better reflect the suitability and access of scientific communications. While many of the CRAS items used in Study 3 align with health literacy goals,^8^ standardized parameters to qualitatively assess PLSAs remain another unmet need.

## Conclusions

PLSAs of scientific content developed by bespoke AI or a bespoke AI-assisted approach take significantly less time and effort to generate, are easier to read, and are deemed higher in quality by end-users compared with those developed by medical writers alone. Furthermore, a bespoke AI approach demonstrates efficiency and quality benefits over a non-bespoke AI approach, underscoring the importance of bespoke workflows, model selection, and prompt optimization in the application of generative AI to PLSA development. Overall, generative AI has the potential to enhance scientific communication by helping medical writers produce PLSAs of scientific content that are fit for purpose. Human review of AI-generated content is still required for quality control based on known AI limitations.

## Supplementary Material

ooaf023_Supplementary_Data

## Data Availability

The data underlying this manuscript will be shared on reasonable request to the corresponding author.
