# Supplementary material for: Using artificial intelligence to expedite and enhance plain language summary abstract writing of scientific content
Source: JAMIA Open. 2025 Apr 3;8(2):ooaf023. doi: 10.1093/jamiaopen/ooaf023 (PMC11967854; doi:10.1093/jamiaopen/ooaf023)
Supplement: ooaf023_Supplementary_Data [file ooaf023_supplementary_data.docx]

# **Supplementary Material**

**Supplementary Table 1. Therapeutic areas and journals used in Study 1**

| **Therapeutic area** | **Number of abstracts** | **Journal** | **Number of abstracts** |
| --- | --- | --- | --- |
| Pain | 1 | *Pain Management* | 1 |
| Respiratory | 1 | *Future Virology* | 1 |
| Immunology | 1 | *Journal of Comparative Effectiveness Research* | 3 |
| Ophthalmology | 1 | *Future Cardiology* | 3 |
| Rheumatology | 1 | *Future Microbiology* | *4* |
| Virology | 1 | *Immunotherapy* | 6 |
| Infection | 1 | *Neurodegenerative Disease Management* | 7 |
| Obesity | 2 | *Future Oncology* | 23 |
| Dermatology | 3 |  |  |
| Fungal disease | 3 |  |  |
| Cardiovascular | 3 |  |  |
| Neurology | 7 |  |  |
| Oncology | 23 |  |  |

PLSA, plain language summary abstract.

All of the PLSAs were published between 2022 and 2023. The predominance of oncology articles is due to the Future Medicine (now Taylor & Francis) publishing guideline.[1] that encourages authors to submit PLSAs.

**Supplementary Figure 1. A PLSA output from the bespoke AI process and revised by a medical writer**

| **PLAIN LANGUAGE SUMMARY**  A study to use smart computers to help write simple summaries of scientific documents  **What is the background or aim of this study?**  Scientists often write summaries of their work, but many are hard for people without medical training to understand. So, researchers wanted to see if using a special kind of computer program called "bespoke artificial intelligence" could help make these summaries easier for people to read. They tested this by using the program to create simple summaries of scientific documents known as ‘abstracts’. These simple summaries are known as plain language summaries, or PLS, for short. They wanted to know if the computer-made PLSs were easier for regular people to understand compared with PLSs written by people.  **What did the researchers do?**  The researchers did three separate studies to see if the computer program they used was helpful. In Study 1, they picked 48 scientific summaries and a PLS for each one that had been written by a person. They then used the computer to make PLSs of the scientific summaries. Then, they checked if the computer-made PLSs were easier to read than the PLSs written by people. Study 2 compared how long it took for people who write medical documents (10 of them) to make PLSs by themselves or using the computer program, and also how much effort it took. Also, some doctors checked if these PLSs were helpful for use in talking with patients. In Study 3, they looked at how easy the PLSs were to read again, this time asking both the people who write medical documents (22 of them) and some patients/patient advocates to give their opinions. They wanted to know if the PLSs were clear and showed understanding and care. So, they did all these studies to see if the computer program was really helpful and if it made the PLSs better for everyone.  **What did the researchers find?**  The researchers found that in Study 1, the PLSs made by the computer program were easier to read than the ones made by people who write medical documents. This was true for all the different ways that they used to check how easy the PLSs were to understand. In Study 2, they saw that the computer program helped the people writing the PLSs save a lot of time. It made making PLSs faster, and it was less work for them. Also, the PLSs made with the computer program were liked more by doctors for explaining things to their patients. In Study 3, they found out again that the PLSs made by the computer were easier to read compared to the ones made by people who are used to writing medical documents. This was the case when they were assessed by both medical writers and patients/patient advocates. So, overall, the computer program was really helpful. It made the PLSs easier to understand, and saved time for the people who wrote them. Also, doctors liked them better for talking to patients, and medical writers and patients/patient advocates thought they were more readable than PLSs written by people.  **What do the findings mean?**  In simple words, using a special computer program helps medical writers make PLSs more quickly and easily. The computer program doesn't make the PLSs harder to understand or less accurate. In fact, it actually makes them just as good or even better than when people write them. So, this computer program helps the writers make PLSs that are well suited for what they're meant for. |
| --- |

PLS, plain language summary; PLSA, plain language summary abstract.

**Supplementary Figure 2. Non-bespoke AI and bespoke AI process block diagrams. The workflow for a researcher or medical writer using the AI-assisted PLSA summarization is as follows: (1) OSA is input as the source document; (2) either the non-bespoke AI or bespoke AI pipeline is run; (3) readability scores are auto-generated; and (4) the PLSA is output as a Word™ document. In the case of the bespoke AI PLSA pipeline, comments from AI regarding validation are included in the output document.**

SUPPLEMENTAL_FIGURE_2_LINK

JSON, JavaScript object notation; LLM, large language model; OSA, original scientific abstract; PLSA, plain language summary abstract

**Supplementary Reference**

1. Future Medicine. Author guide. https://authorservices.taylorandfrancis.com/publishing-your-research/writing-your-paper/how-to-write-a-plain-language-summary/ (accessed 9 July 2024).
